# Unexpected Genetic Twists in Patients with Cardiac Devices

**DOI:** 10.3390/jcm13133801

**Published:** 2024-06-28

**Authors:** Emilia-Violeta Goanta, Cristina Vacarescu, Georgica Tartea, Adrian Ungureanu, Sebastian Militaru, Alexandra Muraretu, Adelina-Andreea Faur-Grigori, Lucian Petrescu, Radu Vătăsescu, Dragos Cozma

**Affiliations:** 1 Doctoral School, “Victor Babes” University of Medicine and Pharmacy, 300041 Timisoara, Romania; goanta.emilia@umft.ro; 2 Cardiology Department, Emergency County Hospital of Craiova, Tabaci Street, Nr. 1, 200642 Craiova, Romania; georgica.tartea@umfcv.ro (G.T.); adrian_909uai@yahoo.com (A.U.); afzanfir@gmail.com (A.M.); 3 Department of Cardiology, “Victor Babes” University of Medicine and Pharmacy, 2 Eftimie Murgu Square, 300041 Timisoara, Romania; petrescu_lucian@yahoo.com (L.P.); dragos.cozma@umft.ro (D.C.); 4 Institute of Cardiovascular Diseases Timisoara, 13A Gheorghe Adam Street, 300310 Timisoara, Romania; andreeaadelinafaur@yahoo.com; 5 Research Center of the Institute of Cardiovascular Diseases Timisoara, 13A Gheorghe Adam Street, 300310 Timisoara, Romania; 6 Department of Physiology, University of Medicine and Pharmacy of Craiova, 200349 Craiova, Romania; 7 Department of Cardiology, Craiova University of Medicine and Pharmacy, 200349 Craiova, Romania; sebastian.militaru@umfcv.ro; 8 Cardiology Department, Clinical Emergency Hospital, 014461 Bucharest, Romania; radu.vatasescu@umfcd.ro; 9 Faculty of Medicine, Carol Davila University of Medicine and Pharmacy, 050474 Bucharest, Romania

**Keywords:** cardiac devices, genetics, sudden cardiac death, pacemakers, defibrillators, resynchronization therapy, loop recorders

## Abstract

**Objective:** To assess the frequency and types of genetic mutations in patients with arrhythmias who underwent cardiac device implantation. **Methods:** Retrospective observational study, including 38 patients with different arrhythmias and cardiac arrest as a first cardiac event. Treatment modalities encompass pacemakers, transvenous defibrillators, loop recorders, subcutaneous defibrillators, and cardiac resynchronization therapy. All patients underwent genetic testing, using commercially available panels (106–174 genes). Outcome measures include mortality, arrhythmia recurrence, and device-related complications. **Results:** Clinical parameters revealed a family history of sudden cardiac death in 19 patients (50%), who were predominantly male (58%) and had a mean age of 44.5 years and a mean left ventricle ejection fraction of 40.3%. Genetic testing identified mutations in various genes, predominantly *TMEM43* (11%). In two patients (3%) with arrhythmogenic cardiomyopathy, complete subcutaneous defibrillator extraction with de novo transvenous implantable cardioverter-defibrillator implantation was needed. The absence of multiple associations among severe gene mutations was crucial for cardiac resynchronization therapy response. Mortality in this group was around 3% in titin dilated cardiomyopathy patients. **Conclusions:** Integration of genetic testing into the decision-making process for patients with electronic devices represents a paradigm shift in personalized medicine. By identifying genetic markers associated with arrhythmia susceptibility, heart failure etiology, and cardiac resynchronization therapy response, clinicians can tailor device choices to optimize patient outcomes.

## 1. Introduction

Arrhythmias and cardiac arrest pose significant challenges in clinical management, requiring various cardiac devices and ablation procedures for treatment [1,2,3,4,5]. Understanding the genetics and clinical factors influencing outcomes is crucial for personalized treatment strategies [6]. Genetic testing has emerged as a valuable tool, providing insights into mutations associated with channelopathies and cardiomyopathies [6,7].

Sudden cardiac death (SCD) represents a devastating outcome, often occurring unexpectedly and leaving a profound impact on affected individuals and their families. While SCD can stem from various cardiac pathologies, channelopathies and cardiomyopathies emerge as significant contributors to this tragic event. Channelopathies encompass a group of genetic disorders characterized by abnormal ion channel function in cardiac cells, leading to arrhythmias and, in severe cases, to SCD. Similarly, cardiomyopathies entail structural abnormalities of the heart muscle, impairing its contractile function and elevating the risk of lethal arrhythmias. 

It is estimated that there are about 7000 single-gene inherited disorders. Many genes responsible for hereditary cardiomyopathies, such as dilated cardiomyopathy (DCM, OMIM #604145), hypertrophic cardiomyopathy (HCM, OMIM #192600), and arrhythmogenic cardiomyopathy (ACM, OMIM #604400), as well as hereditary arrhythmias such as long QT syndromes (LQTS, OMIM #192500), Brugada syndrome (BrS, OMIM #601144), cardiac conduction defects (CCD, OMIM #115080), and catecholaminergic polymorphic ventricular tachycardia (CPVT OMIM #604772), have been identified. 

Congenital LQTS is characterized by a prolonged QT interval on the baseline ECG, usually associated with T-wave abnormalities. Long QT syndrome genes can be classified into three main groups: pathogenic variants that reduce potassium outward currents, pathogenic variants that increase sodium inward currents, and pathogenic variants that increase calcium inward currents. Pathogenic variants related to potassium channels account for the vast majority of *LQTS* cases, with *KCNQ1* and *KCNH2* [8,9] being responsible for 80% of all genetically explained LQTS cases. Currently, the genes with definitive evidence include *KCNQ1*, *KCNH2*, *SCN5A*, *CALM1*, *CALM2*, and *CALM3*. The genes with moderate evidence are *CACNA1C* and *KCNE1*, and testing may be considered in patients with a high probability of diagnosis [10,11].

Brugada syndrome (BrS) is a hereditary disorder characterized by ST-segment elevation in the right precordial leads and malignant ventricular arrhythmias. This syndrome may account for approximately 18–28% of unexplained sudden cardiac arrests. Rare genetic variants in the *SCN5A* gene, leading to the loss of function of the cardiac sodium channel, are found in about 20% of the cases [12,13].

Cardiac conduction disease (CCD) is often age-dependent and is a heterogeneous progressive cardiac conduction disease (PCCD) disorder marked by impaired electrical impulse propagation in the sinoatrial node, atrioventricular (AV) node, and His–Purkinje system. On the surface ECG, sinus bradycardia, sinus pauses, prolonged P-wave duration, AV block, and different degrees of bundle branch block are typical features. The genes involved in CCD are *SCN5A* [14,15] and *TRPM4* [16].

Hypertrophic cardiomyopathy (HCM) is a relatively common hereditary disorder marked by hypertrophy of the left ventricular wall that cannot be attributed to other conditions such as hypertension or valvular heart disease. Typically, the hypertrophy is asymmetric and mainly affects the intraventricular septum. Gene panels that are generally recommended include eight sarcomere genes, including *MYH7*, *MYBPC3*, *TNNI3*, *TNNT2*, *TPM1*, *MYL2*, *MYL3*, and *ACTC1* [17]. This panel typically identifies a disease-causing variant in about 60% of familial cases [18]. 

Dilated cardiomyopathy (DCM) is characterized by the presence of left ventricular or biventricular dilatation and systolic dysfunction. It encompasses a wide range of genetic or acquired disorders. Approximately 100 genes have been identified as potentially related to DCM, with truncating variants in the titin gene (*TTN*) being the most common in DCM, accounting for up to 20% of cases [19]. Genetic testing panels should include the most prevalent genes such as *TTN* and *TNNT2*, as well as genes with prognostic or therapeutic implications, such as *LMNA*, *FLNC*, and *DSP*, or other genes such as *NEXN*, *ACTC1*, and *ACTN2*. The selection of genetic testing panels can be guided by the presence of specific extracardiac phenotypes, such as neuromuscular diseases (e.g., *DMD* and *EMD*), mitochondrial disease (e.g., *NDUFB3*), and congenital syndromes [20].

Arrhythmogenic cardiomyopathy (ACM) is mainly characterized by the replacement of myocardial tissue with fibrous or fibrofatty tissue, which can lead to progressive global or regional ventricular dysfunction with a high burden of ventricular arrhythmias. The recommended genetic test for ACM must include a minimal set of genes that have shown a clinical association with the disease. These genes, by frequency, include *PKP2* (20–45%), *DSP* (2–15%), *DSG2* (4–15%), *DSC2* (2–7%), *FLNC* (3%), *JUP* (1%), *TMEM43* (1%), *PLN* (1%), and *DES* (1–2%) [21]. Initial studies suggested that the *RYR2* gene is part of the genetic basis of ACM. Apart from ACM, *RYR2* mutations have been linked to CCD, DCM, and *CPVT*. 

The identification of individuals at high risk of SCD due to channelopathies and cardiomyopathies poses a considerable clinical challenge. However, advances in genetic testing have revolutionized risk stratification, enabling healthcare providers to pinpoint underlying genetic mutations predisposing individuals to these. 

Genetic testing in descendants of SCD victims plays a pivotal role in unraveling the basis of inherited cardiac conditions. The heritability of certain cardiac disorders, such as long QT syndrome, Brugada syndrome, and hypertrophic or arrhythmogenic cardiomyopathy, underscore the importance of identifying at-risk individuals within affected families [7,22]. 

The genetic make-up of individuals can influence their response to cardiac device therapy. Despite the potential benefits, incorporating genetic testing into routine clinical practice for patients with electronic cardiac devices has some challenges. Issues such as cost, accessibility, and the interpretation of genetic variants need to be addressed. Collaborative efforts between cardiologists, electrophysiologists, and genetic counsellors are essential to overcome this and integrate genetic testing into cardiac care. 

## 2. Materials and Methods

1. Study Design: Retrospective observational study.
2. Inclusion criteria: (I) Patients diagnosed with arrhythmias as a first cardiac event (e.g., ventricular tachycardia, ventricular fibrillation, and 3rd-degree atrioventricular block) who are being treated with cardiac devices, such as pacemakers (PMKs), internal cardioverter-defibrillators (T-ICDs), subcutaneous internal cardioverter-defibrillators (S-ICDs), and undergoing genetic screening; (II) Patients with syncope of unknown cause, who are being treated with loop recorders and undergoing genetic screening; (III) Patients with cardiac resynchronization therapy (CRT) indications, heart failure (HF) belonging to New York Heart Association (NYHA) class II–IV, left ventricular ejection fraction (LVEF) ≤ 35%, QRS complex ≥ 130 ms, left bundle branch block (LBBB) pattern, and optimal pharmacological treatment 3 months prior to CRT, who are undergoing genetic testing.
3. Exclusion criteria: patients with incomplete medical records or missing genetic data.
4. Data collection:
  4.1 Patient demographics: age, gender, and family history of SCD.
  4.2 Clinical characteristics: symptoms, type of arrhythmias, and history of cardiac arrest.
  4.3 Cardiac imaging: (1) Echocardiographic measurements in all patients (valvular regurgitation and ejection fraction (EF)) and (2) Cardiac Magnetic Resonance Imaging (MRI) if available: ejection fraction, fibrosis, or scar.
  4.4 Interventions: type of cardiac device (PMK, ICD, S-ICD, CRT, or loop recorder) and type of ablation if it was performed.
  4.5 Genetic testing used next-generation sequencing panels. The testing focused on channelopathies and cardiomyopathies, used commercially available panels, ranged from 106–174 genes, and were chosen at the discretion of the attending physician.

Depending on availability and local collaboration protocols, certain patients had their genetic testing conducted at the Regional Center of Medical Genetics Dolj (CRGM Dolj). This was performed using the TruSightCardio panel Illumina (San Diego, CA, USA), which included 174 genes. Genomic DNA was isolated from the primary sample using commercial kits (Wizard^®^ Genomic DNA Purification Kit, PureLink™ Genomic DNA, QIAsymphony DSP DNA). Preparation of sequencing libraries was performed according to the manufacturer’s recommendations. The protocol was based on enzymatic fragmentation and selective amplification of target areas. The library obtained was sequenced on the Illumina platform with the aim of obtaining coverage of at least 50× (depth) for germline variants, and at least 98% (coverage) of the targeted areas. Bioinformatics analysis was performed using a bioinformatics solution implemented locally within CRGM Dolj. Variants with convincing evidence of variant/gene–phenotype correlation according to established international databases (OMIM and Clin Var) were especially evaluated. All known modes of transmission for the presumed diseases were taken into account. Mendelian and variants with insufficient criteria for diagnosis were excluded from the report (e.g., heterozygous variants in genes known to be recessive). Bioinformatics tools such as GEMENI, (iGenomes GATK GRCh37 variants nf-core/sarek v2.7.1; Nextflow v21.04.1; BWA 0.7.17; GATK v4.1.7.0) as well as tapes/annovar, could be used for filtering and prioritization. WAS followed the AMCG variant classification, which reflected the probability that a variant is pathogenic in the following sequence: Benign, Likely Benign, Variant with Uncertain Significance (VUS—variant of unknown significance), Likely Pathogenic, and Pathogenic. Only those variants that correlate with the clinical phenotype were reported.

Some patients underwent genetic testing at the Genomic Center of the University of Medicine and Pharmacy Victor Babes Timisoara, utilizing a 174-gene sequencing panel, the TruSightCardio panel from Illumina (San Diego, CA, USA). Target enrichment was conducted with the TruSight Rapid Capture kit (Illumina). Sequence reads were aligned to the human reference genome, hg37, using the Burrows–Wheeler alignment (BWA) tool. Variants identified were annotated using ANNOVAR, as described in previous publications [23].

For some patients, NGS gene panels were utilized at Invitae laboratories (USA) to test for sequence and exon-level copy number variants, as previously described [24,25]. The prescribing physician selected one or more panels among the commercially available NGS panels at their discretion. The basic commercial panel included 106 genes, while larger panels, comprising up to 168 genes, covered a broader set of genes for the Invitae Arrhythmia and Cardiomyopathy Comprehensive Panel, Add-on Preliminary evidence Genes for Arrhythmia and Cardiomyopathy, and Add-on Sudden Unexpected Death in Epilepsy (SUDEP) Genes. Familial screening was offered to relatives of a proband with pathogenic and likely pathogenic variants. The patient and physician chose among the three laboratories based on different turnaround times and reimbursement considerations. The outcomes were device efficacy and device-related complications, arrhythmia recurrence, and mortality. 

### Statistical Analysis

Data are presented as mean ± standard deviation for continuous variables and as proportions for categorical variables. Continuous variables were compared between groups using an unpaired T-test (variables with normal distribution) or Chi-square test. A *p*-value < 0.05 was considered significant. 

All the subjects included in the study gave their informed consent before inclusion. The study was conducted in accordance with the Declaration of Helsinki, and the protocol was approved by the Ethics Committee of our institute (number 46/28.09.2018).

## 3. Results

Thirty-eight patients, 44.5 ± 13.1 y.o. (58% males), were included. All patients received an electronic cardiac device in a tertiary center between 2018–2023. The most frequent presentation that led to the diagnosis was ventricular tachycardia in 34% of the cases, followed by cardiac arrest in 26% of the cases. Demographic, clinical, and echocardiographic parameters are found in Table 1. 

Twenty patients (53%) underwent an MRI scan, and in 70% of cases, extensive fibrosis was identified during the scan. The baseline medication for all the patients is presented in Table 2.

In accordance with the cardiac pathology being the first cardiac manifestation, patients were implanted with an electronic device, as follows: six dual-chamber PMKs, 10 single-chamber ICDs, five dual-chamber ICDs, three subcutaneous ICDs, four loop recorders, 10 CRT,3 CRT-Pacemakers (CRT-P), and seven CRT-D defibrillators (CRT-D). 

Nineteen patients (50%) had a family history of SCD. All the patients were genetically tested, focusing on channelopathies and cardiomyopathies and using commercially available panels, which ranged from 106–174 genes. The results are displayed in graph no. 1. Testing identified pathogenic (P) or likely pathogenic (LP) variants in 27 patients (71%), variants of unknown significance (VUS) in seven patients (18%), and negative results in four patients (11%) (Table 3).

**Figure 1:**
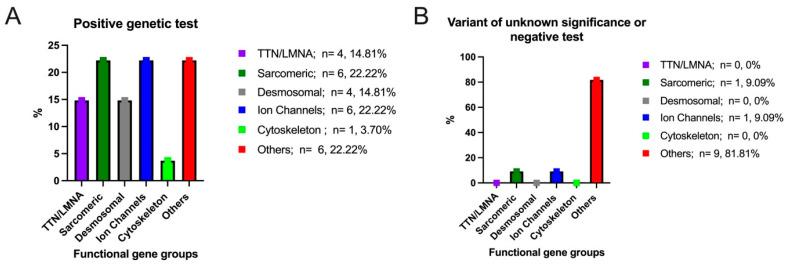
Distribution of positive genetic test (**A**)/variant of unknown significance or negative test (**B**) in the overall study cohort.

According to the recommendations of the American College of Medical Genetics and Genomics (ACMG) [26], the patients included in our study were divided into two groups. The first group included 27 patients with positive genetic tests (pathogenic and likely pathogenic), and the second group included 11 patients either with negative genetic tests or with a variant of unknown significance. We considered *TTN* and *LMNA* as a separate group (14.81% of patients). The following genes were included in the sarcomeric motor genes group: *MYBPC3*, *CTNNA3*, *MYH7*, *TNNI3K*, and *MYLK*. This functional gene group recorded a positive genetic test in 22.22% of patients (Figure 1A). Regarding desmosomal genes, these included *DSP*, *DSC2*, and *PKP2*. This functional gene group recorded a positive test in 14.81% of the patients. Another functional gene group was represented by ion channel genes, which included the following genes: *KCNQ1*, *RYR2*, *SCN5A*, *TRPM4*, and *CACNB2*. This gene group registered positive genetic tests in 22.22% of the patients. In addition, genes from the cytoskeleton-Z-disk gene structural group included DMD, with a positive genetic test in only one patient (3.70%). Patients with mutations in the remaining genes that were screened were categorized into an “other genes” group. These genes include *SGCD*, *TMEM43*, *FBN1*, *A2ML1*, *SOS1*, *AGL*, *NDUFB3*, and *EMD*, and were registered in 22.22% of the patients with positive genetic tests. Regarding the patients with negative genetic tests or with a variant of unknown significance (only 11 patients out of a total of 38 patients), the following genes were identified: *CACNB2* from the ion channel genes group, MYLK from the motor sarcomeric genes group, and *A2ML1*, *SOS1*, *AGL*, and *NDUFB3* from the other genes group (Figure 1B).

**Figure 2:**
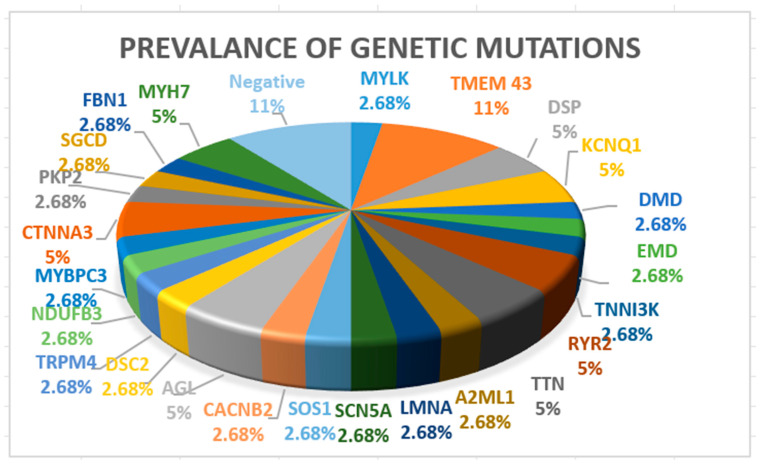
Prevalence of genetic mutations.

Seven patients (18%) had ACM, two patients (5%) had HCM, two had muscular dystrophies (one with Becker and one with Emery Dreifuss tip 1), three patients (8%) had channelopathies (two patients with LQTS type 1 and one patient with BrS syndrome), one patient (3%) had mitochondrial disease, four patients (11%) had CCD, and the rest had DCM. A more detailed image of genetic testing results is presented in Figure 2. 

*TMEM43* mutations (11%) were the most prevalent due to being a disease-causing variant of arrhythmogenic cardiomyopathy. In four patients (11%), genetic testing was negative, two patients had idiopathic ventricular fibrillation, one patient had ACM, and one patient had DCM. In these patients, genetic retesting will be considered using an extensive cardiology panel. 

The average follow-up was 4.7 ± 1.8 years, and the longest follow-up was 6 years. During follow-up, five patients (13%) needed ventricular tachycardia ablation, two patients (5%) underwent atrial fibrillation ablation, and one patient (3%) underwent ganglion denervation.

In ten patients (26%) with DCM, CRT was performed (seven CRT-D, three CRT-P). The assessment of responses to CRT was based on the following criteria [27,28]:

- Clinical response to CRT, defined as improvement in NYHA functional class.
- Echocardiographic response (defined as >5% increase in LVEF and decreased mitral regurgitation degree).

Patients were divided into two groups: super-responders (SRs) and non-SRs (responders and hyporesponders). SR patients were defined as those with a stable ejection fraction (LVEF) ≥ 45%. A detailed comparison regarding SRs and non-SRs characteristics is presented in Table 4.

**Figure 3:**
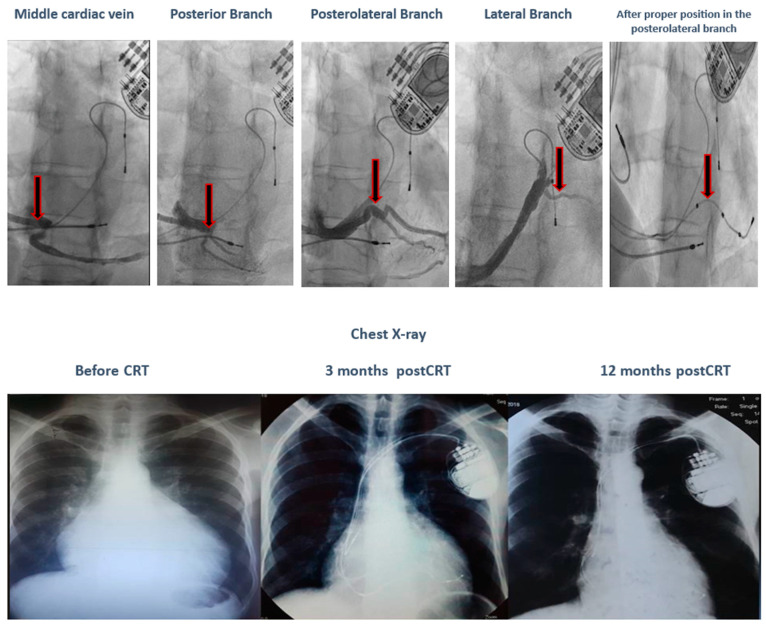
The evolution of a patient with a *TNNI3K* gene mutation after proper positioning of the LV lead in the posterolateral wall.

Comparing the SR vs. non-SR groups, we observe that the SR group has a younger average age, a 100% typical LBBB pattern, a wider QRS complex, and LV leads placed only in posterolateral or lateral positions. During a 12-month follow-up, none of the patients in the SR group experienced severe or moderate mitral regurgitation. In terms of genetic testing, there was a variation of mutations in the SR group, while the non-SR group had two patients with *VUS-AGL* and one patient with both *TTN* and *TMEM43* pathogenic mutations. No deaths were recorded in the SR group. In the non-SR group, there was one cardiac death in the patient with both *TTN* and *TMEM43* mutations due to refractory HF and electrical storm following a severe COVID infection, despite having the clinical and paraclinical criteria of a super-responder (nonischemic, younger age, typical LBBB pattern, wider QRS, and LV lead in the posterolateral position). Additionally, in the SR group, one patient with a *TNNI3K* gene mutation, who met the clinical and paraclinical criteria of a super-responder (nonischemic, younger age, typical LBBB pattern, and wider QRS) on maximal medical treatment, was initially a non-responder due to the inadequate positioning of the LV lead in the anterior wall. After proper positioning in the posterolateral wall, the patient became a super-responder, as illustrated in Figure 3.

An upgrade to CRT-D was necessary in the non-SR group for one patient with a *VUS-AGL* mutation, a possible non-disease-causing variant. This patient had nonischemic DCM with an atypical LBBB pattern and a QRS duration of 140 ms, and was on maximal medical treatment when initially implanted with a CRT-P device. Two years after CRT implantation, due to repeated ventricular tachycardia, a CRT-D upgrade was needed, including replacement of the LV lead from the anterior position to the lateral wall. However, the patient, just like the other one with the same mutation, remained a non-responder. 

**Figure 4:**
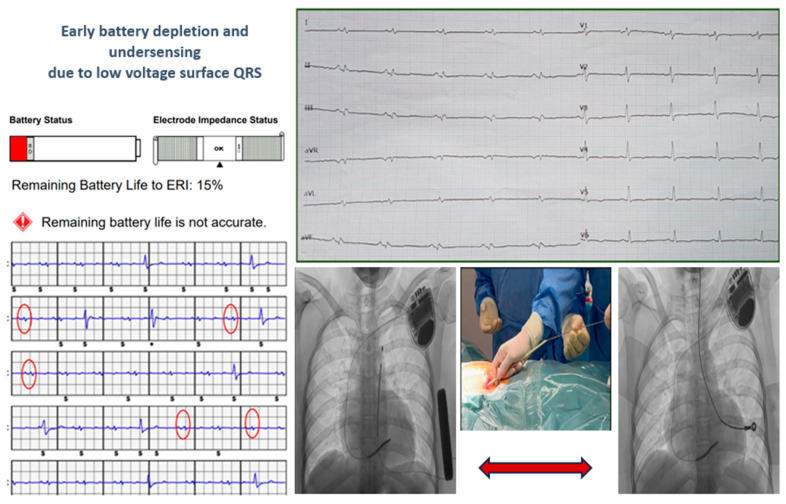
The cases of two young patients with S-ICD who experienced early battery depletion and under-sensing due to low voltage surface QRS. A transvenous ICD (T-ICD) was recommended with complete removal of the S-ICD system. The red circles on the ECG are highlighting QRS underdetection by the S-ICD.

Two young patients, who experienced cardiac arrest due to ventricular fibrillation as their first cardiac event, received a subcutaneous implantable cardioverter-defibrillator (S-ICD). Genetic testing later diagnosed them with arrhythmogenic cardiomyopathy, revealing one *DSP* mutation and one *TMEM43* mutation, both of which are pathogenic. During follow-up, they developed electrical storm due to multiple forms of ventricular tachycardia. Despite undergoing endoepicardial ablation procedures, they continued to experience ventricular tachycardia. Both patients experienced early battery depletion, and the patient with *DSP* mutation also showed under-sensing. Consequently, a transvenous ICD (T-ICD) was recommended with complete removal of the S-ICD system, as shown in Figure 4. 

In the entire group of patients, there were two cardiac deaths, both in individuals with TTN mutations who suffered from refractory heart failure. Despite receiving maximum treatment, their condition progressed rapidly and was fulminant. 

**Figure 5:**
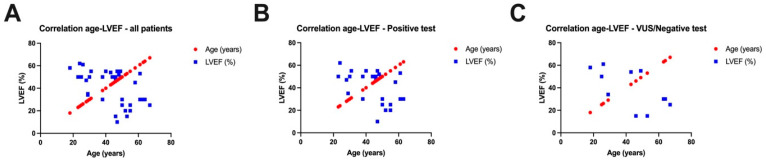
Correlations between left ventricular ejection fraction (LVEF) and age of all patients in the study (**A**), between LVEF and age of patients with positive genetic tests (**B**), and between LVEF and age of patients with a variant of unknown significance or a negative test (**C**).

Assessing the correlation (Figure 5) between the age of the patients included in our study and the left ventricular ejection fraction (LVEF), we observed a negative correlation for all patients included in our study, meaning that the younger the patients, the higher the LVEF values, and the older the patients, the lower the LVEF values (r = −0.5210, *p* = 0.0008). Separating the two groups of patients, we noticed negative correlations both in patients with a positive genetic test (r = −0.4474, *p* = 0.0193) and in patients with a variant of unknown significance or a negative test (r = −0.6354, *p* = 0.0357).

## 4. Discussion 

Genetic testing in patients with electronic cardiac devices holds significant promise for optimizing therapy and improving outcomes. As our understanding of the genetic basis of cardiac disorders continues to expand, integrating genetic information into the clinical decision-making process will become increasingly important. By doing so, clinicians can offer more personalized and effective treatments for patients with pacemakers, defibrillators, and resynchronization therapy devices.

Identifying a specific genetic trait can aid in patient management and guide clinical decisions. Patients with pathogenic *LMNA* variants consistently face a poor prognosis, particularly with a high risk of sudden cardiac death (SCD) due to conduction defects or ventricular arrhythmia. Preventive pacemaker (PM) or implantable cardioverter-defibrillator (ICD) therapy should be considered early for *LMNA* carriers, with ICD implantation algorithms that include pathogenic variant mechanisms (truncating vs. missense variant) due to the higher SCD risk [29,30]. Similarly, a higher risk of SCD is linked to pathogenic variants, especially truncated variants, in the *FLNC*, *DES*, *RBM20*, and *PLN* genes, warranting consideration for preventive ICD implantation in these patients [31,32]. Desmosomal pathogenic variants in individuals with dilated cardiomyopathy or biventricular arrhythmogenic cardiomyopathy are also associated with an increased risk of life-threatening ventricular arrhythmias and SCD [33]. 

Cardiomyopathies can be either inherited and/or acquired [34,35]. They may also be exacerbated by disease modifiers, which are conditions that can worsen or trigger cardiomyopathies (such as many cardiovascular comorbidities). Identifying an acquired cause of DCM does not rule out the presence of an underlying gene mutation; conversely, a genetic variant might require an additional acquired cause to clinically manifest [36,37]. 

Cardiac resynchronization therapy has emerged as a valuable treatment option for heart failure patients [38,39]. As technology advances, genetic testing plays an increasingly pivotal role in tailoring therapies to individual patients’ needs. One critical decision faced by clinicians is choosing between a traditional CRT-P and a CRT-D. 

After the controversial Danish trial, the ICD benefit in primary prophylaxis in patients with non-ischemic cardiomyopathy is debatable in the presence of CRT, and it seems that CRT-P is not inferior to CRT-D [28,40]. 

The data indicate differences within the group we studied. All patients who underwent CRT and genetic testing had non-ischemic cardiomyopathy. Among them, 70% received a CRT-D device following a cardiac arrest caused by ventricular fibrillation or ventricular tachycardia as their first cardiac event, while the remaining 30% received a CRT-P device. One patient with a *VUS-AGL* mutation, a potentially non-disease-causing variant, required an upgrade to CRT-D due to repeated ventricular tachycardia two years post-implantation, which also involved the replacement of the LV lead from the anterior position to the lateral wall. Despite these interventions, this patient, like another with the same mutation, remained a non-responder. Another patient, despite having the clinical and paraclinical criteria of a super-responder (nonischemic, younger age, typical LBBB pattern, wider QRS, and LV lead in the posterolateral position), died due to refractory HF and electrical storm, having both *TTN* and *TMEM43* mutations. It appears that patients with multiple mutations experience more severe disease and exhibit a weaker response to CRT. Furthermore, despite being a nonischemic DCM, a patient with a *TMEM43* mutation, known for its extreme arrhythmogenicity, more often has an unfavorable outcome. 

In human patients, mutations in the nuclear envelope protein TMEM43 are linked to severe diseases, including ACM type 5, a devastating cardiomyopathy that leads to malignant arrhythmias and heart failure. The *TMEM43*-p.S358L mutation has been identified as the genetic cause of an aggressive form of ACM, primarily affecting males. Despite extensive in vivo studies, the pathogenic mechanisms of TMEM43-associated ACM remain poorly understood. Various research groups have developed different models using mice and zebrafish, and induced pluripotent stem cells with *TMEM43* mutations to study ACM [41,42,43]. However, both *TMEM43*-p.S358L knock-in and knock-out mice do not develop a cardiac phenotype under normal conditions [41], suggesting that the pathogenicity of this specific mutation requires enhancement through overexpression or additional genetic, epigenetic, or environmental factors in mice. In the study by Zink et al. [42], the first transgenic zebrafish model expressing two different potential pathogenic variants found in human patients under a heart-specific promoter was created, along with genetic mutants of *TMEM43* in zebrafish. These zebrafish lines were characterized from early embryonic stages to adulthood. The mutant p.S358L *TMEM43* was found to be unstable and partially redistributed into the cytoplasm in embryonic and adult hearts. Additionally, both *TMEM43* variants exhibited cardiac morphological defects at juvenile stages and ultrastructural changes within the myocardium, along with dysregulated gene expression in adulthood. 

Conversely, a patient with a *TNNI3K* gene mutation, who met the super-responder criteria (nonischemic, younger age, typical LBBB pattern, and wider QRS) on maximal medical treatment, was initially a non-responder due to the inadequate positioning of the LV lead in the anterior wall. After proper positioning in the posterolateral wall, the patient became a super-responder. 

Considering our group of patients, in order to become a super-responder, meeting the clinical and paraclinical criteria of a super-responder (nonischemic DCM, younger age, typical LBBB pattern, wider QRS complex, and appropriate LV lead position) is necessary. Additionally, the absence of multiple associations with severe gene mutations is also crucial.

Two patients diagnosed with arrhythmogenic cardiomyopathy, who experienced cardiac arrest due to ventricular fibrillation as their first cardiac event, received subcutaneous implantable cardioverter-defibrillators (S-ICDs). During follow-up, they developed electrical storm due to multiple forms of ventricular tachycardia. Both patients experienced early battery depletion, and the patient with a DSP mutation also showed under-sensing. Consequently, a transvenous ICD (T-ICD) was recommended with complete removal of the S-ICD system. The oldest lead was 6 years old. Multiple simple manual tractions were necessary to release the generator and the proximal part of the lead. For the distal part, a mechanical dilator sheath (yellow sheath, inner ID/OD 8.5/10.7Fr) was used to free the lead. The systems were completely removed without complications. 

Considering this, it might be preferable to implant a T-ICD when arrhythmogenic cardiomyopathy is suspected, even if the patient experienced cardiac arrest due to ventricular fibrillation as their first cardiac event. 

Studies indicate that both T-ICD and S-ICD are effective in detecting and terminating life-threatening arrhythmias in arrhythmogenic cardiomyopathy (ACM); however, the absence of antitachycardia pacing (ATP) in S-ICDs can be a limitation for patients who frequently experience ventricular tachycardia that could otherwise be managed without a shock [44,45]. Guidelines generally support the use of either device in ACM patients, emphasizing personalized treatment based on patient risk profile, lifestyle, age, and arrhythmic burden [2]. As speculated in the literature in cases of severely impaired right ventricular function, a T-ICD is better than an S-ICD [46]. In the remaining cases, an S-ICD could be taken into account, especially in younger patients or those with a higher risk of lead-related complications. 

Regarding mortality, two patients died during follow-up due to refractory heart failure, both of whom had titin mutations. A significant proportion of DCM cases are linked to genetic mutations, with titin mutations being the most common type [47]. Titin is a giant protein that plays a crucial role in the elasticity and stability of cardiac sarcomeres. Studies have shown that patients with this mutation have a worse prognosis, especially a higher incidence of heart failure progression and death, but the extent of the impact can vary based on individual patient factors [48,49]. Early genetic screening and tailored therapeutic strategies are essential in managing patients with *TTN*-related DCM to improve outcomes and reduce mortality, including more aggressive heart failure management and the use of ICD to prevent sudden cardiac death.

The severity and progression of DCM exhibit significant variability among individuals, not only in sporadic cases but also within members of the same family. This variability may be explained by the fact that the clinical phenotype is influenced not only by a single causative gene variant but also by the interaction with common variants in the genome, epigenetic factors, and environmental influences. Patients with DCM who carry pathogenic variants in the *LMNA*, *RBM20*, and *DSP* genes are at higher risk for heart failure progression and may require heart transplantation [33,50].

## 5. Conclusions

Genetic testing has the potential to revolutionize the decision-making process in patients with cardiac electronic devices. By identifying genetic markers associated with arrhythmia susceptibility, heart failure etiology, and CRT response, clinicians can tailor therapy to individual patient needs.

## Figures and Tables

**Table 1:** Demographic, clinical, and echocardiographic parameters. COPD—chronic obstructive pulmonary disease, SD—standard deviation, MR—mitral regurgitation, TR—tricuspid regurgitation.

Male gender, %		22 (58%)
Age, y.o., mean ± SD		44.5 ± 13.1
Main arrhythmia or symptoms leading to cardiac evaluation	Cardiac arrest	10 (26%)
	Ventricular tachycardia	34 (34%)
	Atrial fibrillation	3 (8%)
	3rd degree atrioventricular block	5 (13%)
	Heart failure symptoms	2 (5%)
	Syncope	5 (13%)
Associated pathology, n, %	Hypertension	5 (13%)
	Coronary artery disease	3 (8%)
	Diabetes Mellitus	3 (8%)
	COPD	1 (3%)
LVEF (%)		40.3 ± 14.7
Severe MR, n, %		5 (13%)
Moderate MR, n, %		13 (34%)
Mild MR, n, %		14 (37%)
Severe TR, n, %		1 (3%)
Moderate TR, n, %		8 (21%)
Mild TR, n, %		24 (63%)

**Table 2:** Cardiological medication taken by the patients included in the study; NOAC—non-vitamin K antagonist oral anticoagulant, SGLT2—sodium-glucose co-transporter 2 inhibitors, ACEI—angiotensin-converting enzyme inhibitor, ARNI—angiotensin receptor neprylisin inhibitor.

Medical Treatment	N, %
Bblockers	25 (66%)
Ivabradine	1 (3%)
Class Ic antiarrhythmic	2 (5%)
Class III antiarrhythmic	14 (37%)
NOAC	6 (16%)
Antialdosteronics	16 (42%)
SGLT2 inhibitors	9 (24%)
ARB + ARNI	13 (34%)
ACEI	6 (16%)

**Table 3:** Genetic testing in our group of patients.

Sex	Age (Years)	Gene	OMIM Number	Transcript	Zygosity	Classification
M	49	*TMEM43*	#612048	c.1073C>T (p.Ser358Leu)	heterozygous	P
M	50	*TMEM43&TTN*	#612048&#188840	c.1073C>T (p.Ser358Leu)&c.107635C>T (p.Gln35879*)	heterozygous	P&P
F	28	*DSP*	#125647	Deletion (Exons 7-10)	heterozygous	LP
M	30	*TMEM43*	#612048	c.1073C>T (p.Ser358Leu)	heterozygous	P
M	49	*DSP*	#125647	c.939C>T (Silent)	heterozygous	LP
M	48	*KCNQ1*	#607542	c.691C>T (p.Arg231Cys)	heterozygous	P
F	44	*KCNQ1*	#607542	c.604G>A (p.Asp202Asn)	heterozygous	P
M	29	*DMD*	#300377	Deletion (Exons 45-47)	hemizygous	P
M	24	*EMD*	#300384	c.187+1G>A (Splice donor)	hemizygous	P
F	50	*TNNI3K*	#613932	c.2302G>A (p.Glu768Lys)	heterozygous	P
M	52	*RYR2*	#180902	c.10631C>G (p.Pro3544Arg)	heterozygous	LP
M	50	*TTN*	#188840	c.93166C>T (p.Arg31056*)	heterozygous	LP
F	40	*LMNA*	#150330	c.604G>T (p.Glu202*)	heterozygous	P
F	47	*SCN5A*	#600163	c.5971C>T (p.Arg1991Trp)	heterozygous	LP
F	61	*DSC2*	#125645	c.397G>A (p.Ala133Thr)	heterozygous	LP
M	31	*TRPM4*	#606936	c.1127T>C (p.Ile376Thr)	heterozygous	P
M	63	*MYBPC3*	#600958	c.712C>T (p.Arg238Cys)	heterozygous	LP
F	23	*CTNNA3*	#607667	Deletion (Exon 10)	heterozygous	LP
M	38	*SCN5A*	#600163	c.2989G>A (p.Ala997Thr)	heterozygous	LP
M	46	*RYR2*	#180902	c.5776G>A (p.Val1926IIe)	heterozygous	LP
F	45	*MYH7*	#160760	c.1615A>G (p.Met539Val)	heterozygous	LP
M	47	*PKP2*	#602861	c.1510+1G>T	heterozygous	LP
M	38	*TTN*	#188840	c.69224delA	heterozygous	LP
M	55	*SGCD*	#601411	c.448T>G	heterozygous	VUS
F	61	*TMEM43*	#612048	c.1073C>T (p.Ser358Leu)	heterozygous	P
F	55	*FBN1*	#134797	c.718C>T	heterozygous	P
F	58	*MYH7*	#160760	c.1615A >G	heterozygous	LP
M	46	*MYLK*	#600922	c.3610C>T (p.Arg1204Trp)	heterozygous	VUS
M	53	*A2ML1*	#610627	c.2464G>A (p.Val822Ile)	heterozygous	VUS
M	64	*SOS1*	#182530	c.2165G>A (p.Arg722Lys)	heterozygous	VUS
F	26	*CACNB2*	#600003	c.998C>T (p.Thr333Ile)	heterozygous	VUS
F	63	*AGL*	#610860	c.1333A>G (p.Met445Val)	heterozygous	VUS
F	67	*AGL*	#610860	c.3235C>T (p.Gln1079*)	heterozygous	P
F	25	*NDUFB3*	#603839	c.208G>T (p.Gly70*)	heterozygous	VUS

Gene variants—pathogenic, likely pathogenic, and uncertain significance. M = male, F = female, P = pathogenic, LP = likely pathogenic, VUS = uncertain significance. c.107635C>T (p.Gln35879*) TTN: Exons 153-155 (NM_133378.4) are excluded from analysis. TTN variants are reported in the primary report based on functional effect and/or location. A complete list of variants of uncertain significance, likely benign and benign variants in TTN is available upon request. Variants are named relative to the NM_001267550.2 (meta) transcript, but only variants in the coding sequence and intronic boundaries of the clinically relevant NM_133378.4 (N2A) isoform are reported (PMID: 25589632). c.93166C>T (p.Arg31056*) TTN: Exons 45-46, 147, 149, 164, 172-201 (NM_001267550.2) are excluded from analysis. TTN variants are included in the primary report based on functional effect and/or location. A complete list of variants of uncertain significance, likely benign and benign variants in TTN is available upon request. Variants are named relative to the NM_001267550.2 (meta) transcript. c.604G>T (p.Glu202*) This sequence change creates a premature translational stop signal (p.Glu202*) in the LMNA gene. It is expected to result in an absent or disrupted protein product. Loss-of-function variants in LMNA are known to be pathogenic (PMID: 18585512, 18926329). c.3235C>T (p.Gln1079*) This sequence change creates a premature translational stop signal (p.Gln1079*) in the AGL gene. It is expected to result in an absent or disrupted protein product. This variant has been observed in individual(s) with clinical features of glycogen storage disease type III (PMID: 26885414). In at least one individual the data is consistent with the variant being in trans (on the opposite chromosome) from a pathogenic variant. ClinVar contains an entry for this variant (Variation ID: 551403). c.208G>T (p.Gly70*) This sequence change creates a premature translational stop signal (p.Gly70*) in the NDUFB3 gene. While this is not anticipated to result in nonsense mediated decay, it is expected to disrupt the last 29 amino acid(s) of the NDUFB3 protein. NDUFB11: Deletion/duplication and sequencing analysis is not offered for exon 1. COX10: Deletion/duplication and sequencing analysis is not offered for exon.

**Table 4:** A comparison regarding technical aspects, echocardiography parameters, and genetic mutation in SRs versus non-SRs; LBBB—left bundle branch block, SD—standard deviation, FO—follow-up, super-responders (SRs), non-SRs (responders and hyporesponders), typical pattern—Strauss Criteria, MR—mitral regurgitation.

		SRs (N = 5, 50%)	Non-SRs (N = 5, 50%)
Age, y.o., mean ± SD		51 ± 3.3	63 ± 6.5
LV lead position n, %	Posterolateral	2 (40%)	0 (0%)
	Lateral	3 (60%)	3 (60%)
	Posterior	0 (0%)	1 (20%)
	Anterolateral	0 (0%)	1 (20%)
AV paced interval, mean ± SD		120 ± 7.5	110 ± 10
AV sensed interval, mean ± SD		100 ± 8	90 ± 6.3
LBBB	Typical pattern	5 (100%)	2 (40%)
	Atypical pattern	0 (0%)	3 (60%)
	QRS duration mean ± SD	180 ± 0	140 ± 9.8
LVEF (%) Baseline 12 months FO			
		20 ± 7.1	30 ± 2.4
		50 ± 3.7	35 ± 4.4
Severe MR, n, % Baseline		3 (60%)	0 (0%)
12 months FO		0 (0%)	0 (0%)
Moderate MR, n, % Baseline		2 (40%)	5 (100%)
12 months FO		0 (0%)	4 (80%)
Genetic testing (gene mutation)		*PKP2*	*A2ML1*
		*SGCD*	*AGL*
		*TNNI3K*	*AGL*
		*MYLK*	*MYBCP3*
		*RYR2*	*TTN&TMEM43*

## Data Availability

Data available on request due to restrictions (privacy and ethical reasons).

## Abbreviations

*A2ML1*	Alpha 2 Macroglobulin Like 1
ACM	Arrhythmogenic Cardiomyopathy
ACMG	American College of Medical Genetics and Genomics
*ACTC1*	Actin Alpha Cardiac Muscle 1
*ACTN2*	Actinin Alpha 2
ACEI	Angiotensin-Coverting Enzyme Inhibitor
*AGL*	Amylo-Alpha-1, 6-Glucosidase, 4-Alpha-Glucanotransferase
ARNI	Angiotensin Receptor Neprylisin Inhibitor
ATP	Anti-Tachycardia Pacing
AV	Atrioventricular
BrS	Brugada
*CACNB2*	Calcium Voltage-Gated Channel Auxiliary Subunit Beta 2
*CALM 1*	Calmodulin 1
*CALM 2*	Calmodulin 2
*CALM 3*	Calmodulin 3
CCD	Cardiac Conduction Defects
COPD	Chronic Obstructive Pulmonary Disease
CPVT	Catecholaminergic Polymorphic Ventricular Tachycardia
CRT	Cardiac Resynchronization Therapy
CRT-D	Cardiac Resynchronization Therapy Defibrillator
CRT-P	Cardiac Resynchronization Therapy Pacemaker
*CTNNA3*	Catenin Alpha 3
DCM	Dilated Cardiomyopathy
*DES*	Desmin
*DSG2*	Desmoglein 2
DMD	Dystrophin
*DSP*	Desmoplakin
*DSC2*	Desmocollin 2
EF	Ejection Fraction
*EMD*	Emerin
*FBN1*	Fibrillin 1
*FLNC*	Filamin C
FO	Follow-Up
HCM	Hypertrophic Cardiomyopathy
HF	Heart Failure
ICD	Implantable Cardioverter Defibrillator
*JUP*	Junction Plakoglobin
*KCNQ1*	Potassium Voltage Gated Channel Subfamily Q Member 1
*KCNH2*	Potassium Voltage Gated Channel Subfamily H Member 2
LBBB	Left Bundle Branch Block
LP	Likely Pathogenic
*LMNA*	Lamin A/C
LQTs	Long QT Syndrome
LV	Left Ventricle
LVEF	Left Ventricular Ejection Fraction
MRI	Magnetic Resonance Imaging
MR	Mitral Regurgitation
*MYBPC3*	Myosin Binding Protein C, Cardiac
*MYH7*	Myosin Heavy Chain 7
*MYLK*	Myosin Light Chain Kinase
*MYL2*	Myosin Light Chain 2
*MYL3*	Myosin Light Chain 3
*NDUFB3*	NADH: Ubiquinone Oxidoreductase Subunit B3
*NEXN*	Nexilin F-actin binding protein
NOAC	Non-Vitamin K Antagonist Oral Anticoagulant
NYHA	New York Heart Association
P	Pathogenic
*PLN*	Phospholamban
*PKP2*	Plakophilin 2
*PMK*	Pacemaker
*RBM20*	RNA Binding Motif Protein 20
*RYR2*	Ryanodine Receptor 2
SCD	Sudden Cardiac Death
*SCN5A*	Sodium Voltage Gated Channel Alpha Subunit 5
*SGCD*	Sarcoglycan Delta
*SGLT2*	Sodium-Glucose Co-transporter 2 Inhibitors
S-ICD	Subcutaneous Implantable Cardioverter Defibrillator
*SOS1*	SOS Ras/Rac Guanine Nucleotide Exchange Factor 1
SR	Superresponder
SD	Standard Deviation
T-ICD	Transvenous Implantable Cardioverter Defibrillator
*TMEM43*	Transmembrane Protein 43
*TNNI3K*	TNNI3 Interacting Kinase
*TNNI3*	Troponin I3, Cardiac Type
*TNNT2*	Troponin T2, Cardiac Type
*TPM1*	Tropomyosin 1
TR	Tricuspid Regurgitation
*TRMP4*	Transient Receptor Potential Cation Channel Subfamily M Member 4
*TTN*	Titin
TTN-DCM	Titin Related Dilated Cardiomyopathy
VUS	Variant of Uncertain Significance
